# Biomarkers in Hepatocellular Carcinoma: Current Status and Future Perspectives

**DOI:** 10.3390/biomedicines8120576

**Published:** 2020-12-07

**Authors:** Yasi Pan, Huarong Chen, Jun Yu

**Affiliations:** 1 Institute of Digestive Disease and Department of Medicine and Therapeutics, State Key Laboratory of Digestive Disease, Li Ka Shing Institute of Health Sciences, CUHK Shenzhen Research Institute, The Chinese University of Hong Kong, Hong Kong; yasipan@whu.edu.cn; 2 Department of Anaesthesia and Intensive Care and Peter Hung Pain Research Institute, Institute of Digestive Disease and Department of Medicine and Therapeutics, State Key Laboratory of Digestive Disease, Li Ka Shing Institute of Health Sciences, CUHK Shenzhen Research Institute, The Chinese University of Hong Kong, Hong Kong; hchen2@cuhk.edu.hk

**Keywords:** HCC, biomarker, diagnosis, prognosis

## Abstract

Hepatocellular carcinoma (HCC) is the most common primary liver cancer and one of the leading causes of cancer-related death worldwide. HCC is highly heterogeneous, both within the tumor and among individuals, which is closely related to the HCC surveillance, diagnosis, prognosis, and treatment response. With the advances of next-generation sequencing, the genomic landscape of HCC has been identified which vastly improves our understanding of genetic and epigenetic changes and their interaction during HCC development. In particular, gene mutations, epigenetic modifications, aberrant expression of coding and non-coding RNAs have been extensively explored and many of them are considered as biomarkers for HCC. Most recently, the gut microbiome has been proposed as potential non-invasive biomarkers for HCC diagnosis. In this review, we summarize the current development of HCC biomarkers studies and provide insights on further steps towards precision medicine of HCC.

## 1. Introduction

**Figure 1:**
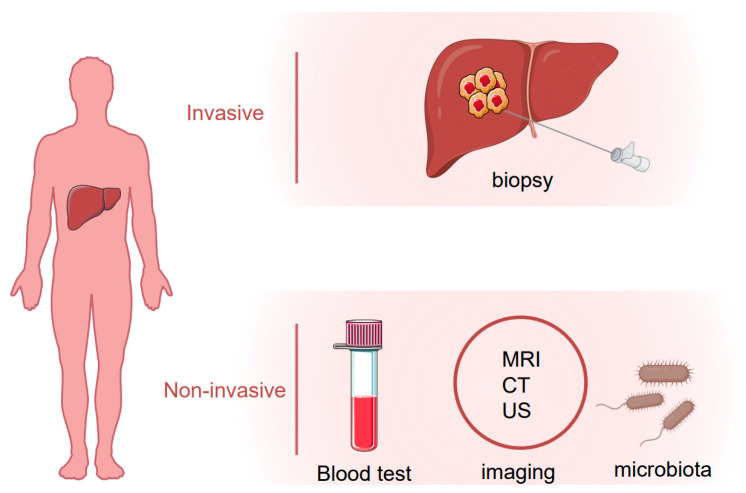
Invasive and noninvasive assessment of hepatocellular carcinoma (HCC).

Hepatocellular carcinoma (HCC) is the most common primary liver tumor and the fourth-leading cause of cancer death worldwide [1]. Several risk factors are known to contribute to HCC development including the hepatitis B or C virus (HBV or HCV) infection, alcohol abuse, obesity and non-alcoholic fatty liver disease (NAFLD) [2]. For HCC patients at an early stage, surgical resection or percutaneous ablation is recommended as a first-line treatment option, and the recurrence rate five years after surgery is around 50% [3,4,5]. On the other hand, more than half of HCC patients were diagnosed with advanced or unresectable disease with a very poor prognosis due to extremely limited therapeutic options [6]. Early detection of HCC using imaging and tumor markers could dramatically improve patient outcomes. For patients with cirrhosis, surveillance of HCC is recommended which endorses significant benefits [7]. Several biomarkers in body fluid samples, e.g., plasma, serum, urine or stool, have been uncovered that could be objectively measured for HCC surveillance and diagnosis. Alpha-fetoprotein (AFP), AFP-L3 and des-γ-carboxy prothrombin (DCP) are the most well-studied and widely used non-invasive biomarkers in HCC. Apart from them, many other molecules such as Glypican 3 (GPC-3), Alpha-l fucosidase (AFU), Golgi protein-73 (GP73) and Squamous cell carcinoma antigen (SCCA), or tumor-associated signatures such as DNA mutation, DNA methylation, micro-RNAs (miRNAs) and long non-coding RNAs (lncRNAs) are under investigation that could be taken into consideration for future clinical practice. In this review, we summarize current HCC biomarker studies, highlighting novel biomarkers and imaging tests that may improve surveillance and diagnosis of HCC in the future (Figure 1).

## 2. Etiologic Factors of HCC

Etiologic agents, e.g., HBV, HCV, alcohol and NAFLD, could lead to chronic liver injury or liver cirrhosis and ultimately HCC, and thus are regarded as the risk factors for HCC. The risk factors of HCC vary in different geographic areas. In China, HBV is accountable for around 54% of HCC cases while 31% of liver cancer cases in Egypt are attributed to HCV infection [8]. As for western countries, NAFLD has emerged as an important cause of HCC in recent years [9]. The development of HCC in NAFLD patients may be associated with excessive body weight, hepatic iron-overload and insulin resistance which could contribute to advanced fibrosis and cirrhosis. Asides from these, gender and age are also thought to be risk factors of HCC. Intriguingly, the prevalence of HCC in males is two to four times more common than in females, a situation called gender disparity [10]. The reasons are complex and could be partially explained by the opposite effects of androgens and estrogen. Estradiol, an estrogen steroid hormone, has been reported to upregulate p53 expression thus suppress HCC [11]. On the other hand, testosterone, the predominant androgen, could promote the hepatocyte cell cycle via cyclin E [11]. In combination with other factors such as HBV and/or HCV infections, age is considered as another risk factor of HCC. A multi-center study across six South American countries involving 1336 patients revealed that nearly 40% of HCC patients with HBV infection at diagnosis were before age 50, while most cases with HCV infection were over the age of 60 [12]. Other environmental factors, such as dietary habits, alcohol consumption and exposure to aflatoxin, are also associated with HCC development.

## 3. Approaches to Identify Potential HCC Biomarkers

Biomarkers are defined as measurable indicators of physiological or pathological processes, or in response to various diagnostic or therapeutic procedures. The development of HCC is characterized by multiple genetic and epigenetic events alterations that run through cancer initiation, promotion and progression. During this process, liver cells are likely to present different molecular signatures, and release certain tumor-associated molecules into body fluid, e.g., blood, urine or stool, that could be monitored for the onset or progression of HCC. The development of detection technology has vastly advanced the development of HCC biomarkers. At present, many biotechnologies have been applied, such as chemiluminescence immunoassay, enzyme-linked immunosorbent assay, immunosensor, proteomics, liquid biopsy, and so on. The advent of Next-Generation Sequencing (NGS) has significantly increased our ability to look into the molecular pathogenesis and heterogeneity of HCC as well as a range of HCC biomarkers, including gene mutations, epigenetic modifications, aberrant expression of coding and non-coding RNAs, and gut microbiome [13,14,15,16,17,18]. An accurate landscape of HCC genetic and epigenetic alterations has been built up with high-throughput analyses of different cohorts which unravels potential biomarkers for monitoring the HCC imitation and progression [15,17]. Meanwhile, a recent study applied genome-wide 5-hydroxymethylcytosines detection using circulating cell-free DNA samples, providing a non-invasive tool in the early detection of HCC [19]. Another new technology, proteome, measuring global protein abundance and post-translational modifications, provides additional biological insights in HCC [14,20]. This method reveals a multi-omics profile of key signaling and metabolic pathways in HCC [20]. The proteomic profiles of tumor-derived extracellular vesicles and particles in human tissues and blood have been well characterized and can serve as reliable biomarkers [21]. Identification of novel non-invasive biomarkers with reliable analytical techniques will shed light on early diagnosis and management of HCC. In the following sections, we will discuss several HCC biomarkers.

## 4. Biomarkers for HCC

### 4.1. Protein Biomarkers

#### 4.1.1. AFP and AFP-L3

Alpha-fetoprotein (AFP) is the most well-studied and commonly used biomarker for the diagnosis and prognosis of HCC [22,23]. AFP is primarily produced by the fetus’s liver and its expression declines rapidly to very a low level by the age of one. However, liver damage or liver cancer can dramatically increase AFP levels in the blood. In a nested case-control study, elevated AFP level could be observed 6 months before the diagnosis of HCC [24], implying that detection of AFP is useful for HCC diagnosis. Current criticisms on the use of AFP mainly focus on its insufficient sensitivity and specificity for early HCC detection if used alone. In addition, increased AFP levels can be found in the setting of cirrhosis patients with active hepatitis, elevated serum alanine aminotransferase (ALT), or non-HCC malignancies [25,26]. To date, AFP detection alone is not recommended for HCC screening. The European Association for the Study of the Liver recommends using liver ultrasound for the surveillance of HCC rather than AFP detection [27]. Nevertheless, the use of AFP is an effective auxiliary diagnostic tool for the detection and surveillance of HCC. In a meta-analysis study comparing the efficacy of ultrasound with or without AFP for early HCC detection (*n* = 2770), Kristina Tzartzeva et al. showed that the use of AFP in combination with abdominal ultrasound can significantly increases the sensitivity of early HCC detection as compared to ultrasound alone (63% vs. 45%) [28]. Moreover, AFP could be used for monitoring HCC progression considering that it promotes tumor proliferation and metastasis [29,30,31]. A meta-analysis consisting of 29 studies and 4726 HCC patients highlighted that AFP level was a potential noninvasive prognosis marker for HCC patients, and AFP Slope > 7.5 ng/mL per month was associated with HCC recurrence post-liver transplantation [32].

AFP-L3, an isoform of AFP, is specific to malignant tumors. The presence of AFP-L3 can serve to identify patients with a high risk of HCC who require increased monitoring. AFP-L3 has been approved by the US Food and Drug Administration (FDA) for assessing the risk of liver cancer. With a cutoff of 1.7%, the use of AFP-L3 demonstrates a better specificity but lower sensitivity for early HCC detection as compared to AFP [33]. Consistently, in a retrospective study recruiting 104 HCC patients with 104 matched non-HCC individuals, the elevation of AFP-L3 was present before the tumor became visible by imaging even though very low AFP levels could be detected, suggesting that AFP-L3 may serve as an early predictive HCC marker [34]. In the future, whether a combination of AFP-L3 and AFP could achieve better diagnostic efficacy for HCC warrants large population-based cohort studies.

#### 4.1.2. DCP

Des-gamma-carboxy prothrombin (DCP), also known as the protein induced by vitamin K absence or antagonist II (PIVKA-II), is a nonfunctional prothrombin [35]. DCP was described as both an autologous growth factor that promotes HCC growth, and a paracrine factor that participates in the crosstalk between HCC and vascular endothelial cells. The biological malignant potential of DCP and its abnormal expression in HCC tissues pinpoints its potential for HCC prediction. In a study involving 1377 HCC patients and 355 patients with chronic hepatitis or cirrhosis, Shinichiro Nakamura et al. compared the diagnostic efficacy of DCP and AFP in discriminating HCC from chronic liver diseases [36]. The results demonstrated that DCP was superior to AFP in detecting large tumors (greater than 5 cm in diameter) [36]. In addition, DCP is a potential prognostic factor for patients with HCC after treatment. In a single-centre retrospective study comprising 412 patients with HBV-related HCC who were treated with radiofrequency ablation, DCP, but not AFP, was found to be an independent prognostic factor for both recurrence-free and overall survival in these patients [37]. The FDA has approved DCP for use in predicting liver cancer. Notably, DCP, AFP and AFP-L3 have been recommended for clinical practice according to Chinese and Japanese guideline [38,39]. However, a recent study carried out in Korea showed that a combination of DCP, AFP and AFP-L3 did not improve the performance for early HCC detection as compared to either AFP or AFP-L3 alone [24]. Further studies are required to evaluate the contribution of DCP for early HCC detection.

#### 4.1.3. GPC-3

Glypican 3 (GPC-3) is a heparan sulfate proteoglycan that plays an important role in cell proliferation and differentiation, and is found to be highly associated with tumor development [40].

GPC-3 has emerged as a potential target for the diagnosis and treatment of HCC recently. GPC3 is rarely expressed in normal hepatocytes or pathological liver cells of hepatitis and cirrhosis. In contrast, GPC-3 is specifically overexpressed in HCC tissues [41]. Consistent results were reported that both GPC-3 mRNA and protein expressions were upregulated in HCC tissues [40,42]. However, the detection of GPC-3 in blood was not as effective as that in tissue biopsies for the diagnosis of HCC [43,44]. By examining serum GPC3 levels in HCC patients using enzyme-linked immunosorbent assay (ELISA), 36.1% to 95% of positive cases could be identified as reported by different studies [45]. Furthermore, serum GPC3 levels were comparable between patients without HCC and those with early HCC [45]. Additional investigations should be carried out to assess the potential of serum GPC3 as non-invasive diagnostic marker for HCC.

#### 4.1.4. AFU

Alpha-l fucosidase (AFU) is a lysosomal enzyme and is reported to participate in the degradation of various fucose-containing fucoglycoconjugates. AFU has been proposed as a potential tumor marker in the diagnosis of HCC. At the cut-off value of 24 U/I, the area under the receiver operating characteristic curve (AUROC) for AFU was 0.83, with sensitivity and specificity of 56.1% and 69.2%, respectively [46]. The diagnostic efficiency of AFU was lower than AFP (cut-off value of 20 ng/mL for AFP) in this study [46]. In contrast, another study involving 1053 HCC patients showed that AFU exerted the same diagnostic power as AFP in both sensitivities (73.52% for AFU vs. 75.01% for AFP) and specificities (76.81% for AFU vs. 82.08% for AFP) [47]. It is worth noting that overexpression of AFP was also observed in other non-HCC diseases such as esophageal squamous cell carcinomas [48] and preeclampsia [49] which could markedly reduce the specificity of AFU for HCC diagnosis.

#### 4.1.5. Other Protein Biomarkers

Proteins that are highly expressed in HCC compared with normal tissues could be promising candidates for HCC detection (Table 1). Golgi protein-73 (GP73, also called Golph2) is a transmembrane glycoprotein primarily expressed in epithelial cells. GP73 has been found upregulated in patients with diverse liver diseases, especially in HCC. In a large cohort study involving more than 4200 serum samples derived from healthy individuals and patients with benign or malignant liver disease, the sensitivity (74.6% for GP73 vs. 58.25% for AFP) and specificity (97.4% for GP73 vs. 85.3% for AFP) of GP73 for detection of HCC were higher than AFP [50]. A consistent result was observed in another study that GP73 had higher diagnostic performance than AFP [51]. Notably, a combination of these two markers could increase the sensitivity for HCC detection to 89.2%, with the specificity of 85.2% [50]. GP73 may also serve as an indicator for the recurrence of HCC given that serum GP73 levels diminished after surgical resection of HCC and rebound after tumor reappeared [50]. Furthermore, serum GP73 level was positively correlated with serum HBV DNA copies and the Child-Pugh score in cirrhotic patients [51]. GP73 is of importance for monitoring the patients with HBV infection who may eventually develop cirrhosis and HCC [51]. GP73 could be used for prediction of HCC in a cirrhotic population. However, in another study, the level of GP73 did not differ among patients with different types of liver disease [52]. Further large-scale and multi-centered studies are needed to evaluate the diagnostic accuracy and surveillance potential of GP73.

Squamous cell carcinoma antigen (SCCA) is composed of two highly homologous proteins SCCA1 and SCCA2 and belongs to the serine protease inhibitor family. SCCA is reported to participate in multiple biological processes such as cell proliferation, resistance to apoptosis, and epithelial-mesenchymal transition. Overexpression of SSCA was identified in HCC tissues at an early stage, indicating that it could be a potential candidate for HCC diagnosis [56]. Following studies were carried out to evaluate the diagnostic value of SCCA [53,55], showing that SCCA complexed with IgM (SCCA-IgM) was useful for assessment of HCC in cirrhotic patients with high sensitivity but poor specificity. A meta-analysis involving 11 studies concluded that both SCCA and SCCA-IgM presented diagnostic value for HCC, with AUROC of 0.8 and 0.77, respectively [57]. Moreover, SCCA can be used to predict the prognosis of HCC patients, thus is recommended to be included in clinical practice in some studies [53].

Others candidates include Apelin [58], β2 microglobulin [59], dickkopf-1 [60], GATA Zinc Finger Domain Containing 1 [61], osteopontin [62] and squalene epoxidase [63] that are reported to have abnormal expressions in HCC as compared to normal control. Although these markers are reported as sensitive biomarkers for HCC prediction, they have not yet been applied in clinical or recommended for use by major professional hepatology societies, probably because of limited sample size, lack of external validation, or sample accessibility, implying the complexity and challenges of biomarker development. Large prospective studies are needed to further validate their performance in HCC diagnosis and prognosis.

### 4.2. DNA Mutation and Methylation Related Biomarkers

The genomic landscape of HCC has been identified with the advances of next-generation sequencing. Over the past decades, a range of single-nucleotide polymorphisms (SNP) has been identified that is correlated with the presence of HCC [64,65]. Take the *EGF* gene as an example. The *EGF* gene polymorphism genotype significantly correlated with *EGF* levels and conferred high risk of HCC development in patients with liver cirrhosis [65]. *EGF* genotype G/G was found to be associated with increased risk of HCC, which may account for the difference in HCC incidence between black and white populations [64]. Besides SNP, telomerase reverse-trancriptase (*TERT*) promoter mutations appeared as an early event of HCC based on the exome sequencing analysis of 243 liver tumors [66]. *TERT* promoter mutations could induce *TERT* transcription or telomerase activation, and promote tumor initiation and progression. In HCC, *TERT* is the most frequent genetic alteration [67]. However, the occurrence of high GC contents in the *TERT* promoter region hinders the development of clinically relevant assays for the detection of *TERT* promoter mutations in patients’ biopsies. In the future, it will be of importance to explore the potential of plasmatic cell-free and tumor DNA (cfDNA and ctDNA) detection of *TERT* promoter mutations for diagnosing early HCC.

Epigenetic alterations such as DNA hypermethylation or hypomethylation are also thought to be early events in hepatocarcinogenesis, thus detection of DNA methylation biomarkers are suitable for detection of HCC. In a large cohort study comprising 1098 patients with HCC and 835 healthy controls, an effective blood-based diagnostic prediction model combining 10-methylation markers (cg10428836, cg26668608, cg25754195, cg05205842, cg11606215, cg24067911, cg18196829, cg2321194, cg17213048 and cg25459300) was established, showing potential for HCC diagnosis with high sensitivity and specificity [68]. Furthermore, the utility of this model demonstrated superior sensitivity and specificity than AFP for HCC diagnosis [68]. Another study comprised of patients harboring different cancer diseases reported that detection of six HCC-specific hypermethylated sites (cg23565942, cg21908638, cg11223367, cg03509671, cg05569109, and cg11481534) could distinguish HCC from other tumor types at very high sensitivity and specificity (92% and 98%, respectively) [69]. Taken together, circulating tumor DNA methylation markers are thought to be reliable for use in the screening, diagnosis, and prognosis of HCC.

### 4.3. miRNA and lncRNAs Related Biomarkers

Noncoding RNAs (ncRNAs) refer to transcripts without protein-coding regions, including microRNAs (miRNAs) and long noncoding RNAs (lncRNAs) (Table 2). miRNAs have been implicated in various biological processes during HCC pathogenesis. Accumulating evidence demonstrated that miRNA can serve as biomarkers for diagnosis, prognosis as well as prediction of therapeutic response of HCC. Detection of miR-221 and miR-101-1 could be used as non-invasive testing for the early diagnosis of HCC among patients with HCV infection [70]. Moreover, miR-939, miR-595 and miR-519 could be used to distinguish HCC from non-HCC among cirrhotic patients, and a combination of these microRNAs was reported to achieve higher AUROC than AFP [71]. Upon combining the AFP, a miRNA panel (miR-122, miR-885-5p, miR-221, and miR-22) was reported capable of distinguishing early HCC from liver cirrhosis, chronic hepatitis C, and healthy subjects, and therefore was recommended for clinical use in early detection of HCC by this study [72]. Moreover, a combination of miR-16, AFP, AFP-L3, and DCP hold great promise in predicting HCC with sensitivity and specificity of 92.4% and 78.5%, respectively [73].

Recently, lncRNAs have also been found to play important roles during HCC development and progression. Several HCC-related lncRNAs have been identified that are dysregulated in HCC tissues, including HULC [74], MALAT1 [75], HOTAIR [76], MVIH [77], and PVT1 [78]. MALAT1 was reported to present a high sensitivity for human HCC prediction, implying that the MALAT1 test could be a potential diagnostic technique [75]. On the other hand, circulating HULC and Linc00152 levels determined by real-time quantitative PCR (qRT-PCR) were found to be higher in the plasma of HCC patients as compared to control in both discovery and validation cohort, with AUROC of 0.78 and 0.85, respectively [79]. Combining HULC and Linc00152 could discriminate HCC from control with AUROC of 0.87 [79]. Thus, serum HULC and Linc00152 may act as novel biomarkers for HCC. Moreover, in combination with AFP and PIVKAII, plasma MALAT1 test could more accurately diagnose HCC as compared to individual parameters, with sensitivity and specificity of 88.6% and 75%, respectively [80]. Nevertheless, larger prospective studies are needed to evaluate the performance of the abovementioned ncRNAs for use in clinical practice.

### 4.4. Gut Microbiome Biomarkers

Gut microbial alteration is likely to contribute to the progression of liver disease and the development of liver cancer through the microbiota-liver axis, implying that detection of HCC-related bacterial could be used as potential non-invasive tools for the prediction of liver cancer (Table 3). In a large clinical cohort study comprising 419 fecal samples from China, 16S rRNA Miseq sequencing was performed and the data analysis using a random forest model identified 30 microbial markers as potential biomarkers for early HCC with AUROC of 0.806 (95% CI, 0.745 to 0.868) and 0.768 (95% CI, 0.679 to 0.857) in discovery and validation cohort, respectively [84]. Compared with the controls, lipopolysaccharide (LPS)—producing bacterial, Klebsiella and Haemophilus, were enriched in patients with HCC; in contrast, Ruminococcus, Oscillibacter, Faecalibacterium, Clostridium IV and Coprococcus were depleted in HCC patients [84]. In another study comparing the gut microbiome between patients with HCC and those with NAFLD-related cirrhosis, the HCC group presented higher enrichment of Bacteroides and Ruminococcaceae but lower abundance of Bifidobacterium compared to the cirrhosis group [5]. Bacteroides and Ruminococcaceae have been reported to associate with immune modulation by inducing proinflammatory cytokines IL18 and IL13 and recruiting activated monocytes and mMDSCs; while deficiency of Bifidobacterium may enhance intestinal and liver inflammation, and subsequently damage the intestinal barrier [85,86]. Apart from gut microbiota, the oral microbiome may also be used as diagnostic biomarkers for HCC [87]. In the future, studies of large-scale are required to further evaluate the role of the gut microbiome as predictors of HCC risk.

### 4.5. Imaging Biomarkers

Unlike other solid cancers, the diagnosis of HCC has relied on the assessment of noninvasive imaging without a regular biopsy confirmation. Meanwhile, chronic liver disease history and biomarkers detections sometimes are taken into consideration for HCC diagnosis. When the noninvasive criteria are not satisfied, needle biopsy may be performed for further confirmation. A systematic review of 47 studies comprising a total of 15,158 patients concluded that patients could benefit from HCC surveillance which has been proved to increase both the detection rate of early-stage tumor and curative treatment rates [88]. Imaging analysis, including computed tomography (CT), magnetic resonance imaging (MRI), ultrasonography (US) or digital subtraction angiography (DSA), plays a critical role in the diagnosis and management of HCC. Currently, patients with cirrhosis or at high-risk of HCC are recommended to take the test of AFP and US every 6 months. US examination is a simple, real-time, non-invasive and sensitive method that can identify the location, size and shape of the liver, thus favors the diagnosis of HCC [89]. In addition, the contrast-enhanced ultrasound (CEUS) has proved itself as a valuable diagnostic tool for HCC prediction with the AUROC of 0.9432 [54]. Due to the limited sample size and potential selection bias in this meta-analysis, further validation is needed to verify the potential of CEUS for HCC diagnosis. MRI is another useful imaging-based biomarker that allows the mapping of the entire liver. In a prospective surveillance study recruiting a total of 407 patients with cirrhosis, the detection rate of HCC by MRI was significantly higher than in the US (86% vs. 27.9%) [90]. About 74.4% of patients with very early stage HCC (a single lesion < 2 cm) were diagnosed and got the chance to receive curable treatment [90]. Taken together, abdominal US is the most commonly method for monitoring patients at high risk of HCC because of its widely availability and low cost. Multiphase CT or multiphases MRI are recommended by the American Association for the Study of Liver Diseases for the diagnosis of HCC.

### 4.6. Biomarker Panels

Because of the limited accuracy for individual marker detection, their combinations have been thoroughly investigated to provide a more precise risk prediction of HCC (Table 4). The GALAD model is a commonly used panel consisting of gender, age, AFP-L3, AFP and DCP [91]. The AUROC of the GALAD in training and validation cohorts that were comprised of 6834 patients was between 0.85 and 0.95. More recently, Shen and colleagues established an online calculator based on serum biomarkers including age, sex, AFP and PIVKA-II, to detect HCC in patients with chronic hepatitis. Their program performed quite well which could accurately predict the presence of HCC in chronic hepatitis patients [92]. Another study developed a logistic regression algorithm, named as Doylestown algorithm, which combined AFP, sex, age, alkaline phosphatase (ALK) and alanine aminotransferase (ALT) and was capable to increase the AUROC of AFP detection for HCC diagnosis [93,94]. In addition, this algorithm could identify high-risk patients 9 months prior to the diagnosis of HCC. Nevertheless, there is a lack of independent validations, and HCC patients of different etiologic agents or ethnic groups should be taken into consideration in future studies.

## 5. Conclusions and Perspectives

Detection of tumor-associated molecular signatures is feasible for monitoring the development and progression of HCC. To date, there are several candidate biomarkers identified for early diagnosis and prognosis prediction of HCC. However, most of them cannot be used in a clinical setting at present, because of the reliability and validity of measurement. The challenges to biomarkers studies remain including incomplete cohort data, selection bias during sample collection and limited sample size for discovery and validation studies. Therefore, larger prospective cohorts with appropriate controls are required for proper validation of identified biomarkers which can be further translated into clinical applications. Moreover, detection technologies and patients’ characteristics should be taken into consideration for clinical biomarker research.

With the advancement of genomics and proteomics, more new biomarkers will be available for early diagnosis and surveillance of HCC. However, HCC is known as a complex disease caused by a variety of risk factors. Thus, it is difficult to use a single biomarker for HCC patients. The development and optimization of biomarkers combination will be more valuable for the early diagnosis and prognosis prediction of HCC. Moreover, demographic characteristics and genetic risks should be considered for the precision screening of HCC. With improved detection technologies and optimization of biomarkers combination, the promise of early detection and treatment of HCC should be realized in the near future.

## Figures and Tables

**Table 1:** Biomarkers for HCC diagnosis.

Biomarker	Samples	Type of Cohort	Sample Size	AUROC or Positive Rate (%)	Sensitivity (%) (95%CI)	Specificity (%) (95%CI)	Cutoff Value	Limitation	Refs
AFP	serum	prospective	689	0.77	62 (48–76)	87 (82–92)	5 ng/mL	Modest accuracy	[24]
AFP-L3	serum	prospective	689	0.73	55 (40–69)	90 (85–94)	4.0%	Modest accuracy	[24]
GPC-3	tissue							Variation among tests	
	mRNA	retrospective	52–105	55.7–100	NA	NA	NA		[45]
	protein		107–757	63.6–91	NA	NA	NA		[51]
GPC-3	serum	retrospective	60–625	31.6–95	NA	NA			[40,45]
DCP	serum	retrospective	689	0.71	48 (33–64)	86 (80–91)	NA	Modest accuracy	[24]
AFU	serum	retrospective	512	0.68	56.1 (NA)	69.2 (NA)	24 U/I	Low accuracy	[46]
GP73	serum	retrospective	60–4217	0.73–0.94	72.4–74.6	61.5–97.4	different cutoff	Modest accuracy	[50,51,52]
SCCA	serum	meta-analysis	12 studies	0.53–0.9	12–84	48–100	different cutoff	Low accuracy	[53,54]
SCCA-IgM	serum	meta-analysis	12 studies	0.66–0.86	51–89	48–78	different cutoff	Low accuracy	[53,54,55]

NA: not available.

**Table 2:** Some biomarkers published in the past five years for HCC diagnosis.

Biomarkers	Testing Method	Type of Cohort	Sample Size	AUROC	Finding	Ref
*CTNNB1, TERT, CDKN2A, SMARCA2* and *HGF*	tissue	retrospective	243	NA	recurrently mutated in alcohol-related HCCs	[66]
*TP53*	tissue	retrospective	243	NA	frequent mutation in HBV-related HCCs	[66]
*TERT*	tissue	retrospective	243	NA	frequent mutation at early stages of HCCs	[66]
Six HCC-specific methylation biomarkers	tissue	TCGA dataset	4389	NA	92% sensitivity and 98% specificity in predicting HCCs	[69]
miR-221	blood	retrospective	115	0.673	56.8% sensitivity and 73.9% specificity for early prediction of HBV-related HCCs	[70]
miR-101-1	blood	retrospective	115	0.763	73% sensitivity and 71% specificity in predicting HCCs	[70]
*PVT1*	blood and tissue	prospective	272	NA	upregulated in HCCs	[78]
*SEPT9*	blood	prospective	334	initial study: 0.944; replication study: 0.930	mSEPT9 test is promising for HCC diagnosis in patients with cirrhosis	[81]
miR-3126-5p	blood	retrospective	155	0.881	downregulated in HCCs	[82]
miR-92a-3p	blood	retrospective	155	0.705	upregulated in HCCs	[82]
miR-107	blood	retrospective	155	0.730	upregulated in HCCs	[82]
*FGF19*	blood	retrospective	304	0.795	cutoff value of 200.0 pg/mL	[83]

NA: Not available.

**Table 3:** Microbiome biomarkers for the diagnosis of HCC.

Biomarkers	Samples	Sample Size	Method	Finding	Diagnosis Potential	Ref
Gut microbiome	faecal	190 (discovery) 131 (validation)	16S rRNA sequencing	*Butyrate-producing bacterial genera ↓ lipopolysaccharide-producing bacterial* ↑	optimal 30 microbial markers achieved AUROC of 80.64% among 75 early HCC and 105 non-HCC samples	[84]
	faecal	61	16S rRNA sequencing	*Bifidobacterium*↓ Bacteroides and Ruminococcaceae↑	correlated with systemic inflammation	[5]
	faecal	223	16s rRNA sequencing	*Bacteroides*↑ *Lachnospiracea incertae sedis*↑ *Clostridium XIVa* ↑	correlated with HBV-related HCC	[18]
Oral microbiome	tongue coat	60	16S rRNA sequencing	*Gammaproteobacteria* ↓ *Bacteroidetes*↓ *Fusobacteria*↑ *Epsilonproteobacteria*↑ *Actinobacteria*↑	Oribacterium and Fusobacterium could distinguish HCC patients from healthy subjects	[87]

↑: upregulated in HCC. ↓: downregulated in HCC.

**Table 4:** Biomarker panels for the diagnosis of HCC.

Biomarkers	Samples	Type of Cohort	Sample Size	AUROC	Finding	Ref
10 DNA methylation markers	blood	retrospective	1933	0.944 in training set 0.966 in validation set	high sensitivity and specificity	[68]
miR-122+miR885-5p+miR-29b+AFP	blood	retrospective	192	1	early detection of HCC	[72]
miR-122+miR-885-5p+miR-221+miR-22+AFP	blood	retrospective	96	0.982	predict HCC in liver cirrhosis patients	[72]
miR+miR-199a-3p+AFP	blood	retrospective	96	0.988	predict HCC in hepatitis C patients	[72]
miR-3126-5p+miR-92a-3p+miR-107+AFP	blood	retrospective	155	0.994	useful for diagnosis of HCC	[82]
gender, age, AFP-L3, AFP and DCP	blood	retrospective	6834	ranging from 0.85 to 0.95 in five international cohorts	useful for diagnosis of HCC	[91]
age, sex, AFP and PIVKA-II	blood	retrospective	2925	0.941 in training set 0.931 in validation set	predict HCC in hepatitis B patients	[92]
